# Association between SARS-CoV-2 infection and anti-apolipoprotein A-1 antibody in children

**DOI:** 10.3389/fimmu.2025.1521299

**Published:** 2025-02-26

**Authors:** Nicolas Vuilleumier, Sabrina Pagano, Elsa Lorthe, Julien Lamour, Mayssam Nehme, Catherine Juillard, Remy Barbe, Klara M. Posfay-Barbe, Idris Guessous, Silvia Stringhini, SEROCoV-KIDS study group, Arnaud G. L’Huillier

**Affiliations:** ^1^ Division of Laboratory Medicine, Diagnostics Department, Geneva University Hospitals, Geneva, Switzerland; ^2^ Department of Medicine, Medical Faculty, Geneva University, Geneva, Switzerland; ^3^ Unit of Population Epidemiology, Department of Primary Care Medicine, Geneva University Hospitals, Geneva, Switzerland; ^4^ Université Paris Cité, Inserm, National Research Institute for Agriculture, Food and the Environment, Centre for Research in Epidemiology and Statistics Paris, Paris, France; ^5^ Geneva School of Health Sciences, University of Applied Sciences and Arts Western Switzerland, Geneva, Switzerland; ^6^ Department of Pediatrics, Gynecology and Obstetrics, Faculty of Medicine, Geneva, Switzerland; ^7^ Division of Child and Adolescent Psychiatry, Department of Woman, Child, and Adolescent Medicine, Geneva University Hospitals, Geneva, Switzerland; ^8^ Division of General Pediatrics, Department of Women, Child and Adolescent Medicine, University Hospitals of Geneva, Geneva, Switzerland; ^9^ Department of Health and Community Medicine, Faculty of Medicine, University of Geneva, Geneva, Switzerland; ^10^ School of Population and Public Health and Edwin S.H. Leong Centre for Healthy Aging, Faculty of Medicine, University of British Columbia, Vancouver, BC, Canada; ^11^ Pediatric Infectious Diseases Unit, Department of Women, Child and Adolescent Health, Geneva University Hospitals, Geneva, Switzerland

**Keywords:** SARS-CoV-2, pediatrics, autoantibodies, apolipoprotein A-1, symptom persistence

## Abstract

**Background and aims:**

Autoantibodies against apolipoprotein A-1 (AAA1) are elicited by SARS-CoV-2 infection and predict COVID-19 symptoms persistence at one year in adults, but whether this applies to children is unknown. We studied the association of SARS-CoV-2 exposure with AAA1 prevalence in children and the association of AAA1 seropositivity with symptom persistence.

**Methods:**

Anti-SARS-CoV-2 and AAA1 serologies were examined in 1031 participants aged 6 months to 17 years old from the prospective SEROCOV-KIDS cohort and recruited between 12.2021 and 02.2022. Four SARS-CoV-2 serology-based groups were defined: “Infected-unvaccinated (I+/V-)”, “Uninfected-vaccinated (I-/V+)”, “Infected-Vaccinated (I+/V+)”, and “Naïve (I-/V-)”. Reported outcomes were collected using online questionnaires. Associations with study endpoints were assessed using logistic regression.

**Results:**

Overall, seropositivity rates for anti-RBD, anti-N, and AAA1 were 71% (736/1031), 55% (568/1031), and 5.8% (60/1031), respectively. AAA1 showed an inverse association with age but not with any other characteristics. The I+/V- group displayed higher median AAA1 levels and seropositivity (7.9%) compared to the other groups (p ≤ 0.011), translating into a 2-fold increased AAA1 seroconversion risk (Odds ratio [OR]: 2.11, [95% Confidence Interval (CI)]: 1.22-3.65; p=0.008), unchanged after adjustment for age and sex. AAA1 seropositivity was independently associated with a 2-fold odds of symptoms persistence at ≥ 4 weeks (p ≤ 0.03) in the entire dataset and infected individuals, but not ≥ 12 weeks.

**Conclusions:**

Despite the limitations of the study (cross-sectional design, patient-related outcomes using validated questionnaires), the results indicate that SARS-CoV-2 infection could elicit an AAA1 response in children, which could be independently associated with short-time symptoms persistence.

## 1 Introduction

Autoantibodies against apolipoprotein A-1 (AAA1) are emerging as an independent risk factor predicting cardiovascular (CV) mortality and morbidity (1–5). The prevalence of AAA1 IgG seropositivity in the general adult population is around 20% (3, 6). So far, few determinants of AAA1 response have been identified, including genetic predispositions (6), previous myocardial infarction (1, 5), autoimmune diseases (7–9), viral infections (human immuno-deficiency virus [HIV] and hepatitis C virus) (10, 11), and niacin therapy (12). The link between mRNA viral infections and AAA1 has been further documented during the COVID-19 pandemic, where SARS-CoV-2 infection was shown to be associated with a significant though transient increase in AAA1 levels and seropositivity (13, 14), possibly explained by the sequence homology between the C-terminus (c-ter; amino-acid region 1140-1170) domain of Spike and the c-ter of apolipoprotein A-1 (apoA-1) (15–18). Furthermore, this SARS-CoV-2-induced autoimmune signature in adults was found to predict persisting symptoms at one year after SARS-CoV-2 infection (14), a feature often retrieved for autoantibodies raised by SARS-CoV-2 infection (19). To date, aside from a small case-control study (20), a thorough evaluation of AAA1 seroprevalence in the pediatric population is still lacking, and it remains unclear whether SARS-CoV-2 infection could induce an AAA1 response in this population. Therefore, we aimed to describe the prevalence of AAA1 IgGs in a large pediatric sample, and to determine whether a SARS-CoV-2-induced AAA1 response could be associated with persisting COVID-19 symptoms up to three months after infection using standardized questionnaires.

## 2 Materials and methods

### 2.1 Study design and data collection

This is an ancillary study from the SEROCoV-KIDS study, a prospective longitudinal cohort study aiming at monitoring and assessing direct and indirect impacts of COVID-19 pandemic on pediatric and adolescent health. We recruited a population-based sample of children and adolescents aged 6 months to 17 years at baseline and living in the canton of Geneva, by inviting eligible participants who were randomly selected from state registries either specifically for this study or for COVID-19 population-based seroprevalence studies conducted by our group (21). The registries were provided by the Swiss Federal Office of Statistics or the Cantonal Office for Population and Migration. Recruitment took place from December 2021 to June 2022. At enrollment, all children were invited to undergo a blood draw to measure anti-RBD and anti-N SARS-CoV-2 antibodies. Parents or legal guardians were requested to complete online and previously validated questionnaires (21, 22), on the Specchio-Hub secured digital platform (21, 22). More specifically, questions referred to whether and when their child(ren) had been infected or vaccinated since the beginning of the pandemic, and whether they suffered from persisting symptoms (lasting ≥ 4 and/or ≥ 12 weeks). Data collection was performed by study coordinators blinded to biochemical results. More details regarding data collection and symptoms evaluation are provided elsewhere (21). In this analysis, we included a subset of participants recruited from December 2021 to February 2022 with both a blood sample and a completed baseline questionnaire (21). During this period in Switzerland the overwhelming Delta variant was swiftly replaced by the Omicron, and systematic genetic characterization was not part of routine procedures.

### 2.2 Anti-SARS-CoV-2 antibody detection

Quantitative Elecsys anti-RBD and semi-quantitative Elecsys anti-N (both measuring total immunoglobulin levels) were tested on the cobas e801 analyzer (Roche Diagnostics, Rotkreuz, Switzerland). Results were reported as concentrations (U/ml) with a manufacturer’s cuf-off >0.8 U/ml considered positive for anti-RBD and as a cut-off index (signal sample/cut-off or signal calibrator), with a cut-off >1 considered positive for anti-N. Quality controls and coefficients of variation for assays are provided elsewhere (23).

### 2.3 AAA1 IgG assessment

AAA1 IgGs were measured as previously described (14), using leftover sera collected for anti-SARS-CoV-2 serologies. OD_405_ was determined at 405 nm, and each specimen was analyzed in duplicate. For each specimen, corresponding non-specific binding was subtracted from the mean OD_405_, and results were expressed as arbitrary units (AU). The previously validated and specified AAA1 IgG seropositivity cut-off was set at an AU (OD_405_) >0.64 corresponding to the 97.5^th^ percentile of the AAA1 IgG distribution retrieved on healthy adult blood donors (1–5, 10, 11).

### 2.4 Study groups definition

Participants were categorized into four groups, defined as follows: Due to faster waning of anti-N antibodies (24, 25), “Infected-unvaccinated (I+/V-)” participants were defined by positive anti-RBD serology and no vaccination history at the time of sampling. “Uninfected-vaccinated (I-/V+)” participants were defined by a vaccination history at the time of sampling along with negative anti-N and positive anti-RBD serologies. “Infected-Vaccinated (I+/V+)” participants were defined by a vaccination history at the time of sampling along with positive anti-RBD and anti-N serologies. Finally, “naïve (I-/V-)” participants were defined by the absence of both infection or vaccination history at the time of sampling, along with negative anti-RBD and anti-N serologies.

For infected participants, time between SARS-CoV-2 positive test (RT-PCR or rapid antigenic diagnostic test [RADT]) and serology was reported. Similarly, for vaccinated patients, time between first SARS-CoV-2 vaccine dose and serology was reported.

### 2.5 Statistical analysis

Continuous variables were expressed by their mean ± standard deviation (SD) or median (interquartile range [IQR]) depending on variable distribution. Categorical variables were presented by their frequencies and relative proportions. For comparisons of continuous variables, parametric Student t-tests and nonparametric Mann-Whitney tests upon variable distribution were used. For categorical variables, either Chi^2^ or Fisher’s exact tests were performed, depending on applicability. Correlations were performed using Spearman correlations. Univariate and multivariate logistic regression models were used to assess the association between AAA1 IgGs seropositivity and symptoms persistence. Adjustment variables were shown to affect either AAA1 response in this cohort (age, anti-RBD and anti-N serology) and/or anti-SARS-CoV-2 response (age, sex). Subgroup analyses were performed in I+/V-, I+/V+, I-/V+ and I-/V-. Results were expressed as odds ratios (ORs) with their 95% confidence intervals (95% CI). Prognostic accuracy of continuous AAA1 IgG levels for symptom persistence was assessed by receiver operating characteristic (ROC) analyses and expressed as the area under the curve (AUC). Sensitivity analyses on participants exposed to natural infection, regardless of vaccination status (I+/V- and I+/V+ combined) were performed. The analyses were performed with SPSS software v23.0 (IBM Corp., Armonk, NY), Statistica™ software (StatSoft, Tulsa, OK, USA) and Excel Analyse-it software ™ (Microsoft Redmond, WA, USA) when appropriate. The graphs were generated using GraphPad Prism 9.0 (GraphPad Software, San Diego, CA, USA). Statistical significance was defined as *P <*0.05 (two-sided).

## 3 Results

### 3.1 Demographics and anti-SARS-CoV-2 antibodies

Of 1070 participants recruited from December 2021 to February 2022, we analyzed 1031 participants who had a completed questionnaire at baseline, a serological test, and enough leftover serum to measure AAA1 IgGs. As shown in **Table 1**, median age was 10 yo (interquartile range [IQR] 7-14) and 524 (50.8%) were female. For further details on pediatric age group distribution within the dataset, please refer to (21). Of all participants, 70% (720/1031) had no past medical history. Among study participants, median time between SARS-CoV-2 positive test (RT-PCR and/or RADT) and blood sampling was 80 days [IQR 27-320]; similarly, median time between first dose of SARS-CoV-2 vaccine and blood sampling was 128 days [IQR 91-176] (**Table 1**). Within the dataset, anti-RBD and anti-N antibodies were positive in 71% (736/1031) and 55% (568/1031), respectively. Anti-N seroconversion was significantly higher in AAA1 seropositive than in AAA1 seronegative ones (71.6 vs 54.1%, p=0.008). I+/V-, I-/V+, I+/V+ and I-/V- participants represented 46% (479/1031), 14% (141/1031), 11% (116/1031) and 27% (278/1031) of the sample, respectively; seventeen participants were not categorized because of uninterpretable serology (weakly positive anti-N, negative anti-RBD). As expected, given national vaccine recommendations at the time of the study, the median age of I+/V- participants (9.0 [IQR 7.0-11.0] was significantly lower than that of the rest of the cohort (12.0 [IQR 8.0-15.0]; p<0.001).

**Table 1:** Patients demographics at baseline.

	Entire cohort n=1031	AAA1+ n=60	AAA1- n=971	p-value	
Age, median (IQR), yrs	10.0 (7.0-14.0)	8.0 (4.0-13.5)	11.0 (8.0-14.0)		**0.002**
Female sex, n (%)	524 (50.8)	36 (60.0)	499 (50.3)		0.145
BMI, median (IQR)	17.3 (15.4-19.6)	16.8 (15.0-19.1)	17.3 (15.4-19.6)		0.146
Comorbidities, n (%)					
asthma	48 (4.7)	2 (3.3)	46 (4.7)		1.000
other chronic lung disease	9 (0.9)	0 (0.0)	9 (0.9)		1.000
endocrine disease	4 (0.4)	0 (0.0)	4 (0.4)		1.000
epilepsy	3 (0.3)	0 (0.0)	3 (0.3)		1.000
physical disability	4 (0.4)	0 (0.0)	4 (0.4)		1.000
blood disease	2 (0.2)	1 (1.6)	1 (0.1)		0.113
rheumatogic disease	1 (0.1)	0 (0.0)	1 (0.1)		1.000
skeletal disease	24 (2.3)	0 (0.0)	24 (2.5)		0.392
inflammatory bowel disease	1 (0.1)	0 (0.0)	1 (0.1)		1.000
other gastro-intestinal disease	5 (0.5)	0 (0.0)	5 (0.5)		1.000
heart disease	3 (0.3)	0 (0.0)	3 (0.3)		1.000
kidney disease	5 (0.5)	0 (0.0)	5 (0.5)		1.000
immunological disease	2 (0.2)	0 (0.0)	2 (0.2)		1.000
Healthy (no past medical history)	720 (69.8)	46(76.7)	674 (69.4)		0.235
SARS-CoV-2 serology and relationship with infection/vaccination					
anti-RBD positive, n (%)	736 (71.4)	44 (73.3)	692 (71.3)		0.731
anti-N positive, n (%)	568 (55.1)	43 (71.6)	525 (54.1)		**0.008**
Time between SARS-CoV-2 positive test and serology, median (IQR), days	80 (27–320)	44 (26–353)	90 (27–316)		0.372
Time between first vaccine dose and serology, median (IQR), days	128 (91–176)	174 (110–201)	127 (90–175)		**0**.223
Exposure category*					
I+/V-	479 (47.2)	38 (64.4)	441 (46.2)		**0.011#**
*persisting symptoms ≥ 4 weeks*	*78 (16.3)*	*11 (28.9)*	*67 (15.2)*	***0.028***	
*persisting symptoms ≥ 12 weeks*	*37 (7.7)*	*4 (18.4)*	*33 (7.5)*	*0.521*	
I-/V+	141 (13.9)	1 (1.7)	140 (14.7)		
*persisting symptoms ≥ 4 weeks*	*18 (12.8)*	*0*	*18 (12.9)*	*1.000*	
*persisting symptoms ≥ 12 weeks*	*9 (6.4)*	*0*	*9 (6.4)*	*1.000*	
I+/V+	116 (11.4)	5 (8.5)	111 (11.6)		
*persisting symptoms ≥ 4 weeks*	*27 (23.2)*	*2 (40.0)*	*25 (22.5)*	*0.330*	
*persisting symptoms ≥ 12 weeks*	*18 (15.5)*	*2 (40.0)*	*16 (14.4)*	*0.171*	
I-/V-	278 (27.4)	15 (25.4)	263 (27.5)		
*persisting symptoms ≥ 4 weeks*	*47 (16.9)*	*5 (33.3)*	*42 (16.0)*	*0.081*	
*persisting symptoms ≥ 12 weeks*	*14 (5.0)*	*2 (13.3)*	*12 (4.6)*	*0.170*	

AAA1, Anti-Apolipoprotein A-1; BMI, body mass index; IQR, interquartile range.

I+/V-, infected/unvaccinated; I-/V+, uninfected/vaccinated; I+/V+, infected/vaccinated; I-/V-, uninfected/unvaccinated.

*17 patients were not categorized because of uninterpretable serology (weakly positive anti-N, negative anti-RBD).

Among those, one was AAA1+, and 16 were AAA1-.

#this refers to the difference in AAA1 seropositivity between exposure categories.Bold values mean significant results.

### 3.2 AAA1 seroprevalence and its relationship with age, comorbidities and SARS-CoV-2

The overall AAA1 seropositivity in the cohort was 5.8% (60/1031). AAA1 seropositive participants were significantly younger than seronegative participants (median age [IQR] 8.0 [4.0-13.5] vs 11.0 [8.0-14.0] years; p=0.002) (**Table 1**). Similarly, there was an inverse correlation between AAA1 levels and age (r=-0.128; p<0.001). AAA1 seropositivity did not significantly differ between participants with (4.5% [14/311] or without comorbidities (6.3% [46/720]; p=0.235). Furthermore, no specific comorbidity was associated with an increased AAA1 seropositivity (data not shown).

**Figure 1:**
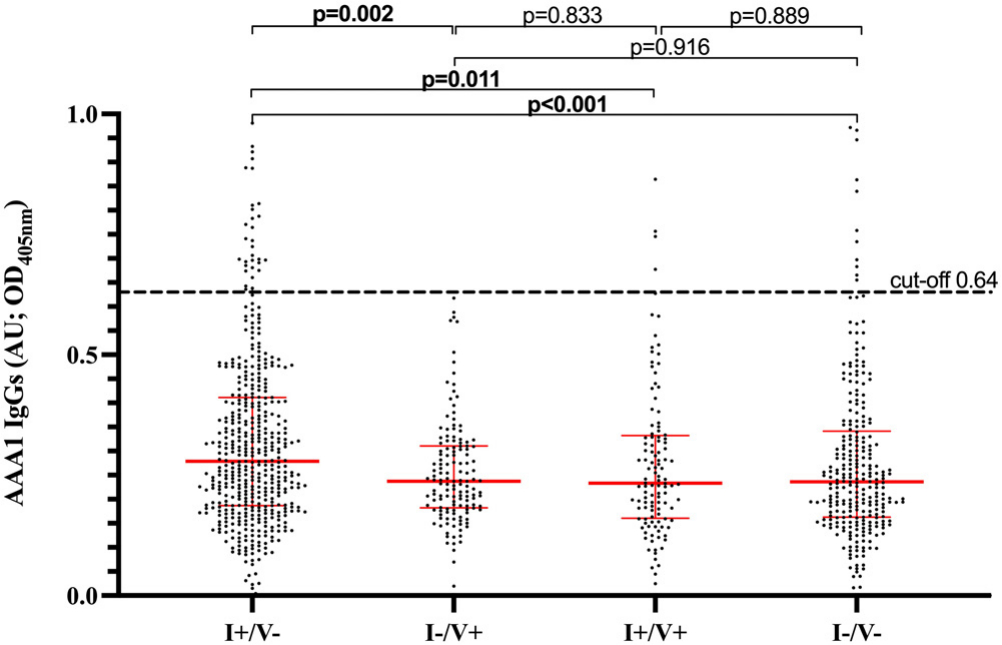
AAA1 IgG levels based on SARS-CoV-2 infection and/or vaccination history. I+/V- (infected/unvaccinated) defined by positive anti-RBD serology and no vaccination history at the time of sampling. I-/V+ (uninfected/vaccinated) defined by a vaccination history at the time of sampling along with negative anti-N and positive anti-RBD serologies. I+/V+- (infected/vaccinated) defined by a vaccination history at the time of sampling along with positive anti-RBD and anti-N serologies. I-/V- (uninfected/unvaccinated) defined by the absence of both infection or vaccination history at the time of sampling, along with negative anti-RBD and anti-N serologies. AAA1, Anti-Apolipoprotein A-1; OD, optical density; AU, arbitrary units.

AAA1 seropositivity was significantly higher in I+/V- (7.9% [38/479]) than I-/V+ (0.7% [1/141]), I+/V+ (4.3% [5/116]) and I-/V- (5.4% [15/278]; p=0.011). Accordingly, there was a significant association between having been infected and being AAA1 seropositive (OR 2.11, 95%CI 1.22-3.65; p=0.0075), which remained unchanged after adjustment for age and sex (OR: 2.11; 95% CI 1.22-3.65; p=0.038) (**Table 2**). Median AAA1 levels were significantly different between the four groups (p<0.0001). More specifically, median AAA1 levels [IQR] were higher in I+/V- (0.28 [0.19-0.41]) than in I-/V+ (0.24 [0.18-0.31]; p=0.002), I+/V+ (0.23 [0.16-0.33]; p=0.011) and I-/V- (0.24 [0.16-0.34]; p<0.001) (**Figure 1**). There was no significant difference between the other groups (**Figure 1**). Overall, the AAA1 seropositivity among children exposed to natural infection, regardless of vaccination status (I+/V- and I+/V+ combined), was 7.2% (43/595). When both I+/V- and I+/V+ groups were combined together to better capture the effect of overall natural SARS-CoV-2 exposure, natural exposure was not associated with a significant increase in the likelihood of AAA1 seropositivity compared to the rest of the cohort (unadjusted OR [95%CI] 1.91 (0.74-4.97); p=0.18; adjusted OR [95%CI] 1.00 (0.35-1.36); p=0.41; **Table 2**). Similarly, when all types of exposures (vaccine and natural) were combined together, there was no association between exposure and AAA1 seropositivity (**Table 2**).

**Table 2:** Nature of SARS-CoV-2 exposure on AAA1 seropositivity.

	Unadjusted OR	Adjusted OR*	Adjusted OR**
	(95%CI)	(95%CI)	(95%CI)
**I+/V-**	**2.11 (1.22-3.65)**	**2.11 (1.22-3.65)**	1.28 (0.45-3.68)
(n=479)	**p=0.0075**	**p=0.038**	p=0.64
**Natural exposure (I+/V- and I+/V+)**	1.14 (0.61-2.04)	1.57 (0.83-2.95)	1.16 (0.56-2.40)
(n=595)	p=0.73	p=0.16	p=0.69
**All exposures (I+/V-, I+/V+ and I-/V+)**	1.91 (0.74-4.97)	1.00 (0.40-1.42)	1.0 (0.35-1.36)
(n=736)	p=0.18	p=0.25	p=0.41

*adjusted for age and gender.

**adjusted for age, gender, anti-RBD and anti-N serologies.

AAA1, Anti-Apolipoprotein A-1; OR, odds ratio; CI, confidence interval.

I+/V-, infected/unvaccinated; I-/V+, uninfected/vaccinated; I+/V+, infected/vaccinated.Bold values mean significant results.

There was no significant difference in time between SARS-CoV-2 positive testing or first SARS-CoV-2 vaccine dose and blood collection between AAA1 positive and AAAA negative patients (**Table 1**). The time between SARS-CoV-2 positive testing (RT-PCR or RADT) and blood sampling was positively correlated with age (r=0.262; p<0.0001), but negatively correlated with AAA1 levels (r=-0.275; p<0.001).

### 3.3 Association between AAA1 and persisting symptoms

There was no difference in AAA1 median (IQR) levels between those with or without symptoms persisting for ≥ 4 weeks (0.26 [0.16-0.40] vs 0.25 [0.18-0.36]; p=0.697) or ≥ 12 weeks (0.26 [0.16-0.38] vs 0.25 [0.18-0.37]; p=0.800).

The proportion of I+/V- participants with symptoms persisting ≥ 4 weeks was higher in AAA1 seropositive (29%) compared to seronegative participants (15%; p=0.028), however no association was found for symptom persistence ≥ 12 weeks (**Table 1**). For the other groups, there was no significant difference in the proportion of participants with symptoms persisting ≥ 4 or ≥ 12 weeks between AAA1 seropositive or seronegative groups (**Table 1**).

Logistic regression models indicated that AAA1 seropositivity was associated with persisting symptoms for ≥ 4 weeks in the whole sample. Even after adjustment for age, sex, anti-RBD and anti-N serology results, AAA1 seropositivity remained associated with a 3-fold risk of symptom persistence ≥ 4 weeks, while only a trend was observed at ≥ 12 weeks (**Table 3**). In subgroup analyses, this effect was confirmed in I+/V-, but disappeared in the vaccinated subgroups and in unexposed individuals (**Table 3**). Sensitivity analyses, including participants with natural exposure (I+/V- and I+/V+ combined) confirmed the significant association between AAA1 seropositivity and symptom persistence for ≥ 4 weeks, but not for ≥ 12 weeks (**Table 3**). However, ROC curve analyses using continuous AAA1 values did not yield any significant results between AAA1 levels and symptom persistence at ≥ 4 or ≥ 12 weeks (**Table 3**).

**Table 3:** Association of AAA1 seropositivity and persisting symptoms according to exposure history.

	Persisting symptoms if AAA1 seropositive					
	Persisting symptoms at 4 weeks			Persisting symptoms at 12 weeks		
	Unadjusted OR	Adjusted OR*	AUC **	Unadjusted OR	Adjusted OR*	AUC **
	(95%CI)	(95%CI)	(95%CI)	(95%CI)	(95%CI)	(95%CI)
**Entire cohort**	**2.32 (1.30-4.15)**	**2.58 (1.42-4.69)**	0.51 (0.46-0.57)	1.93 (0.91-4.34)	**2.27 (1.01-5.08)**	0.50 (0.43-0.58)
(n=1014)	**p=0.004**	**p=0.001**	p=0.28	p=0.08	**p=0.04**	p=0.43
** I+/V-**	**2.27 (1.07-4.80)**	**2.30 (1.07-4.96)**	0.52 (0.44-0.59)	1.45 (0.48-4.34)	1.46 (0.48-4.43)	0.52 (0.42-0.62)
(n=479)	**p=0.03**	**p=0.03**	p=0.33	p=0.50	p=0.50	p=0.74
** I+/V+**	2.29 (0.36-14.5)	1.99 (0.30-12.96)	0.50 (0.37-.64)	3.96 (0.61-25.57)	3.25 (0.49-21.86)	0.53 (0.35-0.70)
(n=116)	p=0.38	p=0.47	p=0.97	p=0.15	p=0.22	p=0.78
** I-/V+**	NA#	NA#	NA#	NA#	NA#	NA#
(n=141)						
** I-/V-**	2.63 (0.86-8.09)	3.24 (0.98)	0.50 (0.40-0.61)	3.22 (0.65-15.90)	4.59 (0.84-25.02)	0.54 (0.37-0.72)
(n=278)	p=0.09	p=0.05	p=0.96	p=0.15	p=0.07	p=0.63
** Natural exposure (I+/V- and I+/V+)**	**2.17 (1.08-4.31)**	**2.28 (1.13-4.62)**	0.51 (0.44-0.57)	1.66 (0.67-4.14)	1.78 (0.70-4.51)	0.51 (0.42-0.60)
(n=595)	**p=0.02**	**p=0.02**	p=0.81	p=0.27	p=0.22	p=0.82

*adjusted for age, gender, anti-RBD and anti-N serologies.

** AUC computed using continuous AAA1 values.

#only one participant AAA1+ in this subgroup.

AAA1, Anti-Apolipoprotein A-1; OR, odds ratio; CI, confidence interval; AUC, area under the curve; NA, not available.

I+/V-, infected/unvaccinated; I-/V+, uninfected/vaccinated; I+/V+, infected/vaccinated; I-/V-, uninfected/unvaccinated.Bold values mean significant results.

## 4 Discussion

This study first shows that the prevalence of AAA1 seropositivity in a representative sample of the pediatric population is three to four times lower than what has been found in the general adult population (5.8 vs 20%) (3, 6). However, as opposed to adults, we could not detect any association with chronic pediatric comorbidities. This might be related either to power issues due to the relatively small number of individuals with comorbidities in our dataset, or simply to the fact that AAA1 levels can be influenced by numerous diseases specific to the adult population (1, 5, 10, 11, 14). Unexpectedly, AAA1 levels were significantly and inversely associated with age, while no relation between age and AAA1 levels has been established so far in adults (1, 2, 5, 13, 14).Two major factors can explain such findings. Firstly, SARS-CoV-2 infected children in our dataset were younger than SARS-CoV-2 uninfected children. Secondly, there was a positive correlation between age and time between SARS-CoV-2 positive test and blood sampling which, given transient nature of AAA1 kinetics after SARS-CoV-2 infection (14), could potentially explain that some older children in the dataset seroreverted against AAA1.

Secondly, our results indicate that SARS-CoV-2 infection can be associated with an AAA1 response in children, similar to what has been previously reported in adults (13, 14). The fact that the significant association between natural infection and AAA1 seropositivity was lost when vaccinated participants are considered together with those naturally exposed could be viewed as an indication that natural SARS-CoV-2 infection only induces an AAA1 response. This hypothesis is supported by the fact that I-/V+ pediatric participants displayed significantly lower AAA1 seroprevalence (and levels) than most other groups, especially I+/V- participants (0.7% vs 7.9%), highlighting the absence of association between mRNA vaccination and AAA1 seropositivity in children. Due to the existence of scarce but equivocal data about the possible impact of mRNA vaccine on AAA1 response in adults (26), our current observations in children are reassuring, even if the present study design does not allow us to formally confirm our hypothesis.

One of the most interesting findings of our work is possibly the association between AAA1 seropositivity and persisting symptoms. Indeed, our data show that in the pediatric population, AAA1 seropositivity was an independent predictor of persisting symptoms for ≥ 4 weeks in the whole sample, but only a non-significant trend for symptom duration >12 weeks. Upon subgroup analysis, we were able to document that this association was mostly driven by I+/V- participants, confirming our previous findings in the adult population that natural AAA1 seropositivity following SARS-CoV-2 infection is an independent predictor of persisting symptoms (14). Sensitivity analyses, including participants with previous natural infection, regardless of vaccination status, also confirmed this association. The inability to replicate these findings using ROC curves requires further investigations and can be explained by mutually non-exclusive hypotheses, including a type I error, the suboptimal sensitivity of ROC curves analyses (27), a possible threshold-effect, or a non-linear association with hazards, as recently reported(4).

This study has several limitations. The first one is related to our cross-sectional study design, with retrospective assessment of SARS-CoV-2 infection and persistence of symptoms, which does not allow us to assess causality. The second limitation is related to the fact that data regarding persisting symptoms were retrospectively collected using validated questionnaires instead of independently adjudicated outcomes. Third, the AAA1 seropositivity cut-off has not been validated in children; even though we could question whether this this limits the validity of the results, it is important to clarify that commonly used serology and auto-antibody assays do not have specific pediatric cut-offs. Fourth, results regarding the four groups might have been limited by underpowering and potential adjudication bias given the methodology used for their respective definition. Fifth, given the wide range of time between positive SARS-CoV-2 test and serology in our dataset, potential AAA1 waning might have underestimated potential associations. Then, questionnaires might have missed some rare complications or complications not yet reported at the time of study design, such as myositis and neuropsychiatric syndromes (28–30), which is an additional limitation. Finally, due to the study design which had to comply with routine diagnostic procedures at the time of the pandemics, the lack of sequencing of positive specimens impedes the exploration of SARS-CoV-2 variants-specific impact on AAA1 response. As the immune response strongly differs between variants (31), the AAA1 response might have differed as well.

In conclusion, our study highlights a lower AAA1 seroprevalence in the general pediatric population compared to adults, without any obvious associations with chronic comorbidities. Furthermore, this study shows that natural SARS-CoV-2 infection is an independent predictor of AAA1 seropositivity, which was found to be independently associated with persisting symptoms for one month. While this confirms what has been previously documented in adults, understanding the mechanistic of AAA1-induced autoimmune phenomena, and if and how these results could be of any use for long COVID management remain to be determined.

## Data Availability

The raw data supporting the conclusions of this article will be made available by the authors, without undue reservation.

## Group members of SEROCoV-KIDS study group

Andrew S. Azman, Antoine Bal, Rémy P. Barbe, Hélène Baysson, Aminata R. Bouchet, Paola D’Ippolito, Roxane Dumont, Nacira El Merjani, Francesco Pennacchio, Natalie Francioli, Idris Guessous, Séverine Harnal, Julien Lamour, Arnaud G L’Huillier, Andrea Loizeau, Elsa Lorthe, Chantal Martinez, Shannon Mechoullam, Mayssam Nehme, Klara M. Posfay-Barbe, Géraldine Poulain, Caroline Pugin, Nick Pullen, Viviane Richard, Deborah Rochat, Serguei Rouzinov, Khadija Samir, Stephanie Schrempft, Silvia Stringhini, Stéphanie Testini, Deborah Urrutia Rivas, Anshu Uppal, Charlotte Verolet, Jennifer Villers, Guillemette Violot, María-Eugenia Zaballa.
